# Generation of ESTs for Flowering Gene Discovery and SSR Marker Development in Upland Cotton

**DOI:** 10.1371/journal.pone.0028676

**Published:** 2011-12-06

**Authors:** Deyong Lai, Huaizhu Li, Shuli Fan, Meizhen Song, Chaoyou Pang, Hengling Wei, Junjie Liu, Dong Wu, Wenfang Gong, Shuxun Yu

**Affiliations:** 1 College of Plant Science and Technology, Huazhong Agricultural University, Wuhan, Hubei, People's Republic of China; 2 Key Laboratory of Cotton Genetic Improvement of Ministry of Agriculture, The Cotton Research Institute, Chinese Academy of Agricultural Sciences, Anyang, Henan, People's Republic of China; 3 College of Agronomy, Northwest A&F University, Yangling, Shanxi, People's Republic of China; 4 College of Agriculture and Biotechnology, Zhejiang University, Hangzhou, Zhejiang, People's Republic of China; Kyushu Institute of Technology, Japan

## Abstract

**Background:**

Upland cotton, *Gossypium hirsutum* L., is one of the world's most important economic crops. In the absence of the entire genomic sequence, a large number of expressed sequence tag (EST) resources of upland cotton have been generated and used in several studies. However, information about the flower development of this species is rare.

**Methodology/Principal Findings:**

To clarify the molecular mechanism of flower development in upland cotton, 22,915 high-quality ESTs were generated and assembled into 14,373 unique sequences consisting of 4,563 contigs and 9,810 singletons from a normalized and full-length cDNA library constructed from pooled RNA isolated from shoot apexes, squares, and flowers. Comparative analysis indicated that 5,352 unique sequences had no high-degree matches to the cotton public database. Functional annotation showed that several upland cotton homologs with flowering-related genes were identified in our library. The majority of these genes were specifically expressed in flowering-related tissues. Three Gh*SEP* (*G. hirsutum* L. *SEPALLATA*) genes determining floral organ development were cloned, and quantitative real-time PCR (qRT-PCR) revealed that these genes were expressed preferentially in squares or flowers. Furthermore, 670 new putative microsatellites with flanking sequences sufficient for primer design were identified from the 645 unigenes. Twenty-five EST–simple sequence repeats were randomly selected for validation and transferability testing in 17 *Gossypium* species. Of these, 23 were identified as true-to-type simple sequence repeat loci and were highly transferable among *Gossypium* species.

**Conclusions/Significance:**

A high-quality, normalized, full-length cDNA library with a total of 14,373 unique ESTs was generated to provide sequence information for gene discovery and marker development related to upland cotton flower development. These EST resources form a valuable foundation for gene expression profiling analysis, functional analysis of newly discovered genes, genetic linkage, and quantitative trait loci analysis.

## Introduction

Cotton is the leading agronomic fiber and oilseed crop in the world. *Gossypium hirsutum* L. is a primary cultivated allotetraploid species (known as upland or American cotton) and has a tetraploid genome (AD; 2n = 4× = 52) [1], [2]. The products from this species include fibers and seeds that have a variety of uses. Cotton fibers sustain one of the world's largest industries, namely textiles, and cottonseeds are widely used for food oil, animal feeds, and industrial materials. In addition to its economic importance, upland cotton has attracted considerable scientific interest among plant breeders, agricultural scientists, taxonomists, developmental geneticists, and evolutionary biologists because of its unique reproductive developmental aspects and speciation history [3]–[5].

0The flowering behavior (initiation and development) of higher plants is one of the most important aspects during plant development. When plants undergo an initial period of flowering, the vegetative shoot apical meristem is transformed into an inflorescence meristem. Inflorescence meristems then respond to both environmental and endogenous flowering signals to give rise to floral meristems, which go on to produce the various types of floral organs including the familiar sepals, petals, stamens, and carpels [6]. In all seed crops, the transition from vegetative to reproductive growth is one of the most important developmental switches because it determines the production of dry matter in the life cycle. Shifting the seasonal timing of reproduction is a major goal of plant breeding efforts to produce novel varieties that are better adapted to local environments and climate change [7]. Recent evidence suggests that genes controlling the timing of flowering affect hybrid vigor and are thus likely to impact yield [8]. Upland cotton is a natural perennial with an indeterminate growth habit that has been adapted to annual cultivation by plant breeders because of its economic importance. The time of the first flowering has been used to determine the earliness, which is a basic breeding objective in upland cotton [9]. Understanding the molecular mechanism of flowering and flowering habits would greatly accelerate molecular breeding research of upland cotton.

The entire genomic sequence is not available for cotton species, but a large number of genome resources have been developed for cotton, especially for upland cotton. These include polymorphic markers [10], genes for important agriculture traits [11], expressed sequence tags (ESTs) [12], large-insert bacterial artificial chromosome libraries [13], and genome-wide, cDNA-based or unigene EST–based microarrays [14]. Analysis of ESTs is one of the most efficient approaches to provide transcriptome resources, and this method is complementary to a whole-genome sequencing project [15]. A large number of ESTs has been produced from cDNA libraries constructed using mRNA isolated from different organs of upland cotton including the root, stem, seedling, leaf, fiber, ovule, and boll. The overwhelming majority of these EST resources is from developing fibers or fiber-bearing ovules, whereas only a minority is from non-fiber and non-ovule organs. The availability of such EST resources has allowed rapid progress in gene discovery and gene identification in these tissues during development [16]. However, flowering-related EST resources from upland cotton are scarce. This hinders both the identification of functional genes and the construction of framework genetic linkage maps related to flower development. Therefore, we constructed a normalized and full-length cDNA library for efficient generation of comprehensive EST resources from upland cotton shoot apexes, squares, and flowers. These ESTs will be used as resources for gene discovery and will form a foundation for cloning the full-length sequences of the genes. They will also provide microarray elements for gene expression profiling and assist in developing molecular markers such as simple sequence repeats (SSRs) that are potentially useful for genetic linkage mapping and quantitative trait locus analysis in upland cotton. In this study, we describe the generation and analysis of 22,915 ESTs that have been deposited in GenBank under the accession numbers HO089234 to HO112148, and we identify several flowering-related genes and the development of novel EST-SSR markers in upland cotton.

## Results

### Generation of flowering-related ESTs in upland cotton

A normalized and full-length cDNA library from shoot apexes, squares, and flowers of upland cotton was constructed to generate ESTs. The insert sizes ranged from 500 to 3,000 bp with an average size of 1,200 bp. A total of 24,283 cDNA clones were randomly isolated and sequenced from the 3′ end using the primer M13-R. All raw EST sequences were trimmed of vector sequences, the poly (A) tail, and low-quality sequences and filtered for minimum length (100 bp). This resulted in 22,915 high-quality ESTs with an average length of 528.4 bp. These EST resources are suitable for gene discovery and molecular marker identification related to flower development of upland cotton.

### EST assembly and analysis

**Figure 1:**
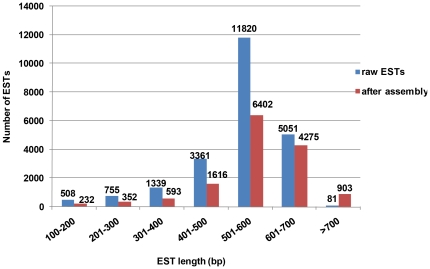
Sequence length distribution of the ESTs before and after assembly.

To produce non-redundant EST data and to improve sequence accuracy for further analysis, the newly generated ESTs were assembled into clusters by sequence identity. The 22,915 upland cotton ESTs generated from 3′-end sequencing resulted in 14,373 unigenes including 4,563 (31.7%) contigs that consisted of two or more ESTs and 9,810 (68.3%) singletons (Table 1). The average length of the unigene sequences was 562.7 bp (range 100–1,498 bp). Figure 1 compares the distribution of the sequence length before and after sequence assembly. Of these unigenes, 13,611 (94.7%) assembled cDNA sequences had open reading frames (ORFs) that were longer than 100 bp, and the longest ORF of each sequence was selected for further analysis. The average ORF length was 283.6 bp (range 100–1,107 bp).

**Figure 2:**
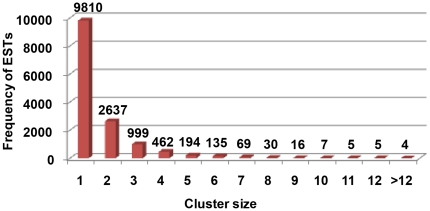
Frequency and distribution of upland cotton ESTs among assembled contigs.

In our study, the full-length cDNA library was normalized to subtract highly expressed genes and to isolate ESTs corresponding to rare or low-expression genes. Of the 4,563 contigs, 2,637 (57.8%) contained two ESTs, 999 (21.9%) contained three ESTs, 462 (10.1%) contained four ESTs, 194 (4.3%) contained five ESTs, 135 (3.0%) contained six ESTs, and relatively few sequences (3.0%) contained more than six ESTs (Figure 2). On average, each contig was assembled from 2.9 sequences due to a few highly redundant ESTs, and the unigene mean size was only 1.6 sequences. This showed that the quality of the normalization of this library was very good.

**Table 1:** Summary of the ESTs from 24,283 cDNA clones in upland cotton.

Feature	Value
Total ESTs	24,283
High-quality ESTs	22,915
Contigs	4,563
ESTs in contigs	13,015
Singletons	9,810
Unique sequences	14,373
Redundancy (%)	37.3
Average length of unigene sequences (bp)	562.7

### Comparison to previous cotton ESTs

It is interesting that the library contains novel unique sequences that do not match any sequences in the existing databases. To estimate the contribution of our library, the 22,915 EST sequences from upland cotton were compared against the ESTs and unigenes already available in the DFCI (Dana-Farber Cancer Institute) database, which contains 351,954 cotton ESTs and 2,315 ETs totally assembled to 117,992 unique sequences. Approximately 37.2% of the unique sequences generated in this study were not highly homologous to existing cotton ESTs and unique sequences of the CGI (Cotton Gene Index) database. Thus, our library added 5,352 cotton unique sequences, which therefore represented a new transcript resource.

### Putative functional annotation and categorization of unique ESTs

To assess their putative identities, all distinct ESTs were subjected to BLAST sequence similarity searches against the NCBI nt (non-redundant nucleotide database), nr (non-redundant protein database), and the SwissProt database, which contain all the nucleotide or protein sequences submitted to the public databases. In the NCBI nt database, 10,780 (75.1%) had significant matches against cotton and other species with the mean E-value cutoff set to 7.8e-08. Within these significant matching sequences, only 1,571 sequences had hits with previously published *Gossypium* nucleotide sequences, including just 1,327 upland cotton nucleotide sequences. The NCBI nr database is commonly used as the principal target database to search for homologous proteins. Most of the upland cotton unique sequences (84%) had the best matches with proteins in the NCBI nr database. However, 2,301 sequences had no hits. Furthermore, the majority of the BLAST hits were recorded for *Ricinus* (26.4%), *Vitis* (24.5%), and *Populus* (23.4%), whereas only 886 (7.3%) of the entries were identified for the cotton. In total, 8,130 (80.1%) of the unigenes matched genes with a search against the SwissProt database. The best hits in BLASTx searches were mainly to *Arabidopsis* (3845 hits, 47.3%) and rice (364 hits, 4.5%).

**Figure 3:**
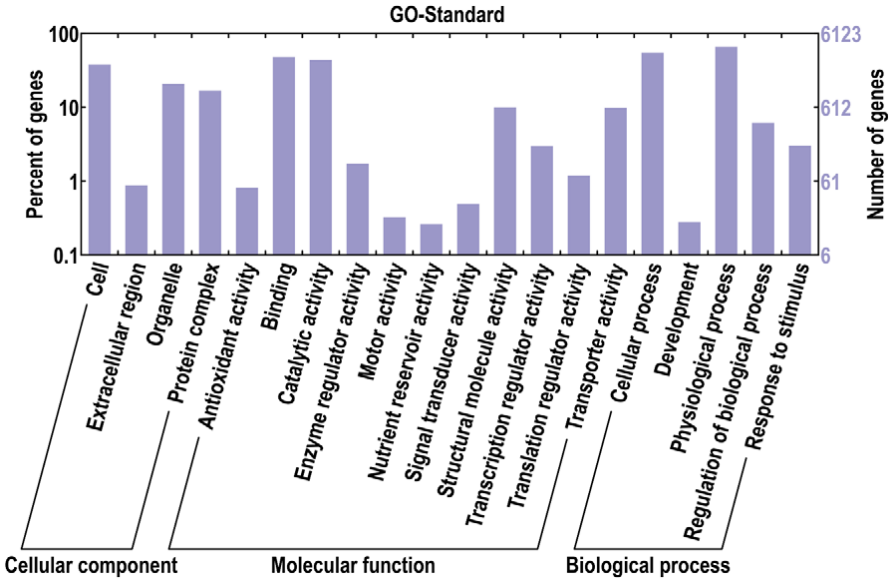
Distribution of EST consensus sequences in the main GO functional classes. The bar chart shows the distribution of ESTs among the three GO categories: cellular location, molecular function, and biological process.

Gene ontology (GO) analysis has been widely used to characterize gene function classification [17]. A total of 6,131 (43%) unigenes were annotated and divided into three GO categories. In total, 3,361 were categorized under the “cellular component” category, 8,527 were categorized under “molecular function”, and 5,546 were categorized under “biological process”. Within the “cellular component” category, 49.6% belonged to “cell”, followed by 27.3% to “organelle”, 22.0% to “protein complexes”, and 1.2% to “extracellular regions”. In the “molecular function” category, the most GO terms (40.2%) were included in “binding”, followed by “catalytic activity” (36.6%), “structural molecule activity” (8.4%), “transporter activity” (8.2%), and “transcription-regulator activity” (2.5%). In the “biological process” category, “physiological processes” and “cellular processes” had 50.6% and 42.1% of the GO terms, respectively. They were followed by “regulation of biological processes” (4.7%), “response to stimuli” (2.3%), and “development” (0.2%) (Figure 3). These results indicate that the unique transcripts are involved in different categories covering many aspects of tissue development and offer a good representation of the upland cotton genome.

### Identification of flowering-related genes in upland cotton

A list of upland cotton homologs of flowering-related genes was found in our library from functional annotations of unique ESTs as described above (Table S1). The 34 candidate genes that were identified included flowering determination genes, floral meristem identity genes, and floral organ development genes. Most of the putative flowering-related genes (26/34) identified were involved in flowering determination process. This is the first stage of reproductive growth initiation, and it is regulated by environmental factors such as light, temperature, and endogenous cues to determine the flowering time [18]–[21]. Four unigenes were identified as a best match with the floral meristem identity genes from *Arabidopsis thaliana* and *Solanum tuberosum*. These transcripts were floral meristem identity genes that confer floral identity to the developing floral primordia[22]–[25]. The ABC model of flower organ identity provides a framework for understanding the specification of flower organs in diverse plant species [26]. In our library, four flower organ identity genes were annotated for several unigenes. The homologs for all the flower development stages can be found in our library.

### Tissue expression patterns of flowering-related ESTs

**Figure 4:**
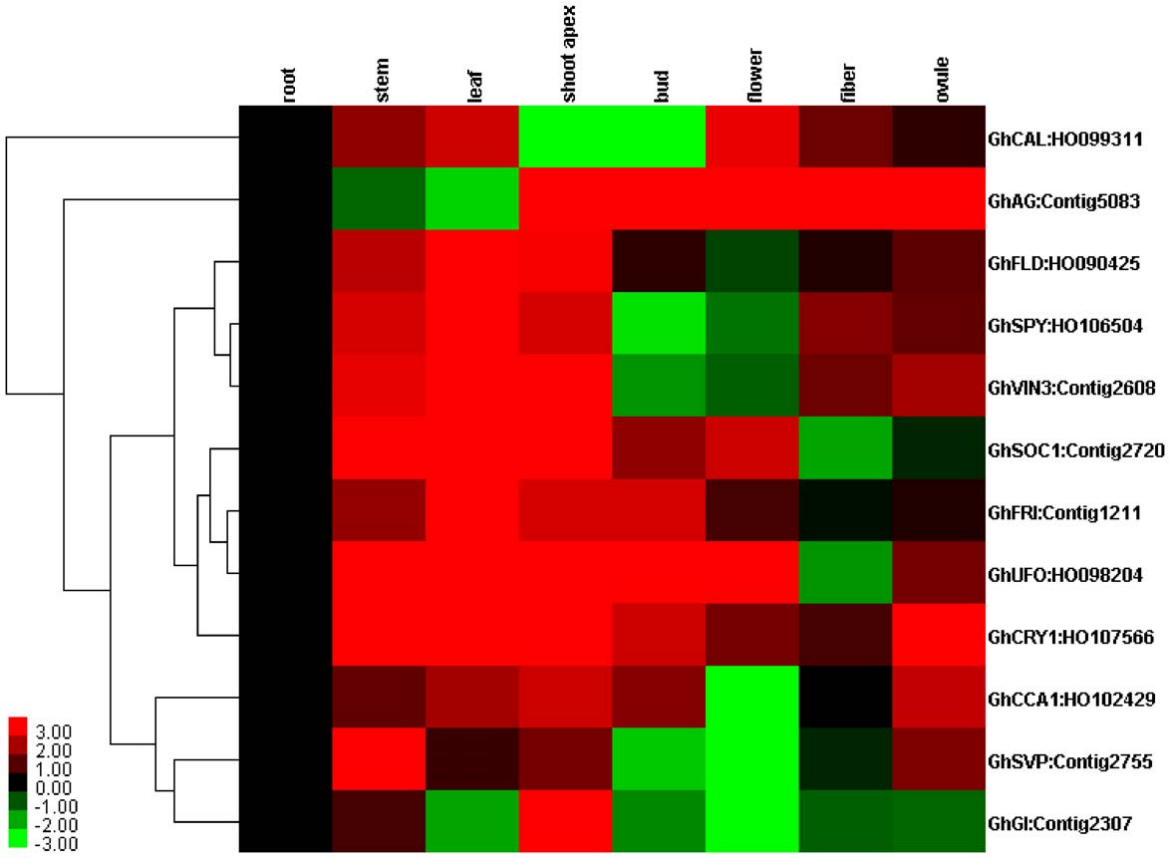
Tissue expression patterns of 12 putative flowering genes from upland cotton. qRT-PCR was used to evaluate the relative levels of flowering-related ESTs in different tissues (root, stem, leaf, shoot apex, bud, flower, fiber, and ovule) with endogenous 18S and root samples used for reference. The patterns were clustered and viewed using Gene Cluster and TreeView software (Stanford University).

The 34 putative flowering-related ESTs that were generated in this study show high identities with the flowering-related proteins of some species. To validate the differential gene expression results and obtain more refined gene expression data, 12 transcripts were randomly selected from Table S1. Gene-specific primers were designed for these transcripts (Table 2) to analyze expression using qRT-PCR (Figure 4). Most of these ESTs were expressed in limited tissues and were not expressed ubiquitously in all seven tissues examined. However, some unigenes such as Contig2307, HO102429, and HO107566 were highly expressed in the shoot apex, and HO099311 accumulated in and was expressed in the flower. Furthermore, some transcripts such as Contig2608, HO106504, HO090425, and Contig1211 were highly expressed specifically in the leaf and shoot apex, whereas HO098204 accumulated in the stem and square. The majority of these transcripts were thus expressed mainly in the tissues that were chosen to construct the library (shoot apex, square, and flower). These results suggest that these transcripts are expressed in flower-related tissues during the floral meristem transition or during flower development.

**Table 2:** Primers used in gene-specific qRT-PCR of flowering genes.

Gene name	Primer sequence (5′-3′)
Gh*CCA1*	F:TCATTGTAGGGATGCGGCTGTT
	R:CGAGTTGCTGGTGGATGGGTT
Gh*CRY1*	F:ATGCCCAGATCATTTATCCATAAG
	R:AATAATGGGAATTGGCTCTGG
Gh*FRI*	F:TCTCCGACTGCTTTATCAGGTTCTGC
	R:GATCCTGGCGAGTTGACCGAGTTA
Gh*VIN3*	F:GAGATGCTGGATCAGAAATGAAGA
	R:GGAGAACGACAAGACGAGGAAT
Gh*FLD*	F:GCCGAAGTCAATTTCTTCCTCA
	R:CCAATGTTCCATACTCTGACCCTAA
Gh*SOC1*	F:AACGCCTTCTTTAGCAAACCAT
	R:ACCCTACAAGCAGGCAAGTGA
Gh*UFO*	F:GCCACTGCTGCCAAGGTAAG
	R:CGGACAAGGGACTGCTGTTT
Gh*GI*	F:AGAGGGACCACGGAAACCA
	R:CAGCACATCAGTCCTTCGCAAT
Gh*CAL*	F:TCTCCACGAAAGTTCCTCAA
	R:GGTTCTGAATCACAGGCAAA
Gh*SVP*	F:GAACTTCTTGATTGACCTGCTCTAA
	R:GTGCTGGACTGCATGAAGGATAT
Gh*SPY*	F:TCCGTCACAGACAGGTGATTTAG
	R:CAATGTCGGTGTCAGTCTTCTCA
Gh*AG*	F:CCAGCATGTGCCTGTTTGTATT
	R:ATTGTCTTCTCCAACCGTGGTC
Gh*SEP1*	F:TCCGCTCCACCAAGACCC
	R:GACAAAGCCCTGTTAGTTTCCAT
Gh*SEP2*	F:AACCAACCCATCAGCCTCAG
	R:GGTAGCCATCCCGTCATGTAAT
Gh*SEP3*	F:AATGAAGTTGGATGGAAGTGGTC
	R:AGGTGGTGGATGGTTGTAT
*18S*	F:AACCAAACATCTCACGACAC
	R:GCAAGACCGAAACTCAAAG

### Cloning of upland cotton *SEPALLATA* homologous genes; sequence, phylogenetic, and expression analysis

**Figure 5:**
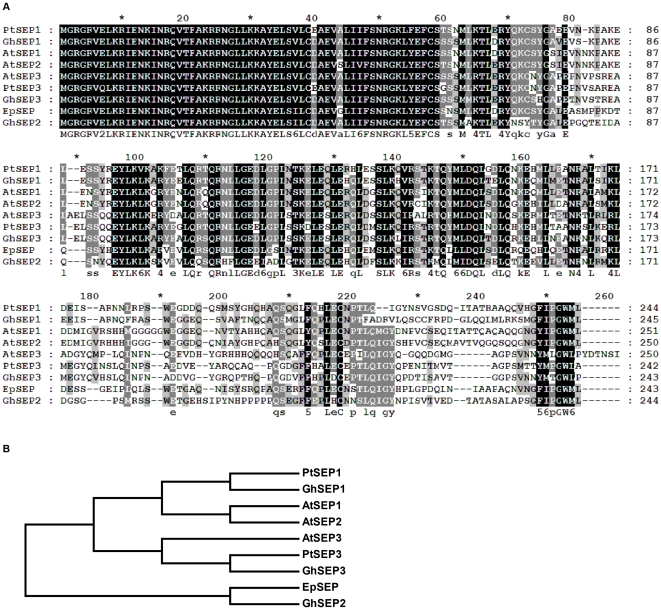
Multiple sequence alignment of Gh*SEP* and other plant *SEP* proteins. A: Multiple protein sequence alignment of Gh*SEPs* with other plants: *Populus SEP1/2* (XP_002330922), and *SEP3* (AAO49811), *Arabidopsis SEP1* (NP_568322), *SEP2* (NP_186880) and *SEP3* (NP_850953), and *Euptelea SEP* (ADC79707). The MADS-box domain was highly conserved among the *SEP* sequences. B: A phylogenetic tree of these plant *SEP* proteins constructed with MEGA 4.1.

**Figure 6:**
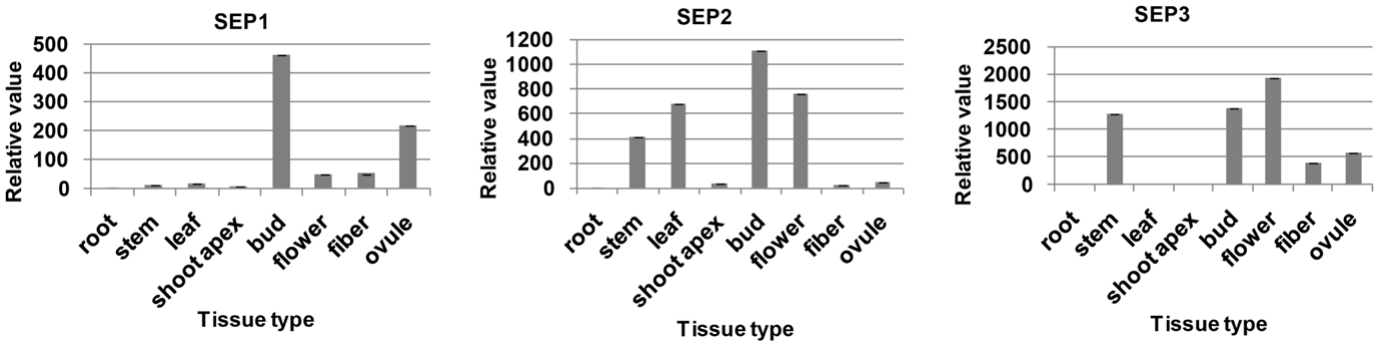
Expression analyses of Gh*SEP* genes in cotton tissues. The expression levels in the different tissues were quantified using qRT-PCR as described above.

To validate that our full-length library was an efficient method for rapid functional gene discovery of upland cotton genes, three members of the *SEPALLATA* (*SEP*) gene family were cloned and analyzed. The *SEP* genes encode transcription factors of the MADS-box gene family, which determine floral organ identity [27], [28]. The *Arabidopsis SEP* proteins were used as a query to search our EST database with tBLASTn. Three unique full-length sequences were found in upland cotton. These sequences were named Gh*SEP1* (JF271884), Gh*SEP2* (JF271885), and Gh*SEP3* (JF271886). The cDNAs of Gh*SEP1–3* have ORFs of 738 bp, 735 bp, and 732 bp, encoding proteins of 245, 244, and 243 amino acid residues, respectively. The Gh*SEP* genes share high sequence homology at the nucleotide level (71–77% identity) in the coding region. The BLAST analysis showed that proteins derived from these cDNAs are homologous to the *SEPs* from *Arabidopsis*, *Populus*, and *Euptelea* with identities of 66–84% at the amino acid level (Figure 5). Multiple sequence alignment of Gh*SEPs* and their homologous proteins revealed that cotton Gh*SEP* proteins also contain the conserved MADS-box domain (Figure 5). Tissue expression patterns of the Gh*SEP* transcripts in various cotton tissues were analyzed using qRT-PCR (Figure 6). Gh*SEP1* was preferentially expressed in the square, whereas Gh*SEP2* and Gh*SEP3* transcripts mainly accumulated in the square and the flower. Flowering-related genes could be identified from our library using the homologous sequence search; however, they could not be found from the unigene annotation.

### Characterization of SSR marker sequences

**Figure 7:**
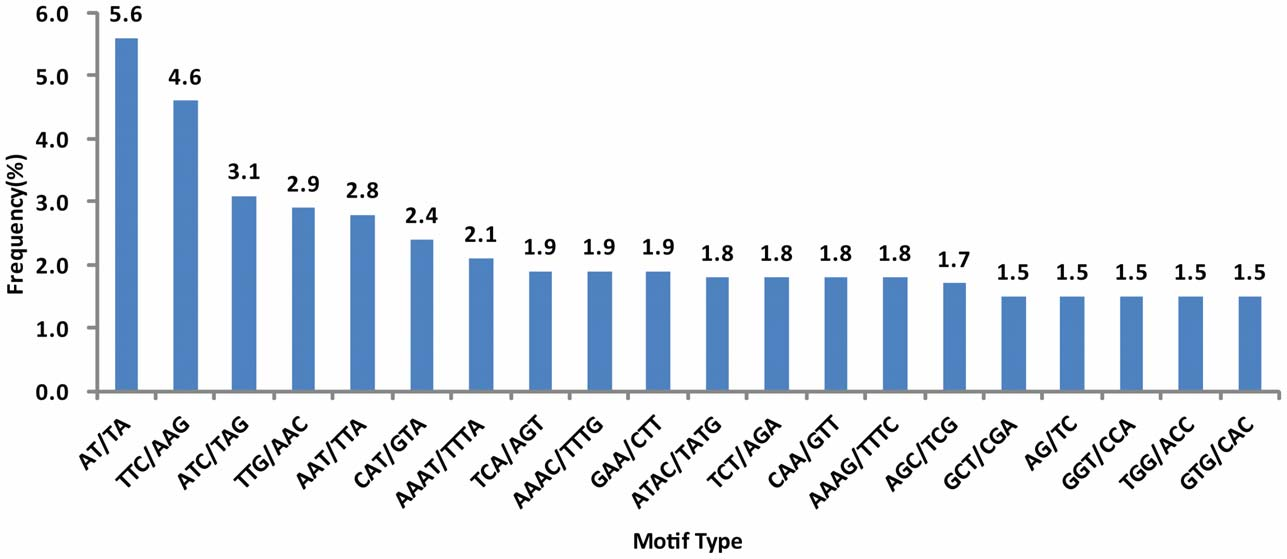
Frequencies of different repeat motifs in EST-SSRs from upland cotton.

A high-density genetic map is an important tool for representing the cotton genome structure and evolution. EST-SSRs are functional markers whose polymorphisms may cause changes in gene function and lead to phenotypic variation. In recent years, many SSRs from different cotton genomes or tissues have been developed and utilized. To develop new EST-SSRs, the 14,373 unigenes were examined using the software SSRIT. In total, 2,295 putative microsatellites were detected from 1,964 sequences and then compared with all 16,162 publicly available SSR markers in the Cotton Marker Database (CMD) [29]. The resulting dataset included 720 new SSRs in these flowering unigenes, and 670 new EST-SSRs were developed from the 645 sequences with long flanking sequences necessary for the design (Table S2). The EST-SSR repeat types are summarized in Table 3. Among these 670 new EST-SSRs primer pairs, the most abundant repeat type was trinucleotide repeats (322, 44.7%), followed by tetranucleotide repeats (221, 30.7%) and hexanucleotide repeats (74, 10.3%). The motif type AT/TA was the highest frequency of 5.6% followed by the motif TTC/AAG (4.6%), ATC/TAG (3.1%), TTG/AAC (2.9%), and AAT/TTA (2.8%) (Figure 7).

**Table 3:** Features of microsatellite markers identified in upland cotton flower ESTs.

Motif length	No. of EST-SSRs	Frequency (%)
Dinucleotide repeats	65	9.0
Trinucleotide repeats	322	44.7
Tetranucleotide repeats	221	30.7
Pentanucleotide repeats	74	10.3
Hexanucleotide repeats	38	5.3

### The *Gossypium* species EST-SSR transferability

To investigate whether the potential SSR loci mined were true-to-type and could be used for genetic analysis, 25 EST-SSR primer pairs (Table S3) were randomly selected and verified in *Gossypium* species. Only one of these primers (CCRI005) could not amplify any fragment in upland cotton. The primer CCRI019 produced amplicons, but they were larger than the expected size. The remainder of the primer pairs generated clear DNA bands with the expected sizes. Thus, 92.0% (23 of 25) of EST-SSR primers could be used to analyze genetic diversity. The 23 markers verified were used to screen 17 *Gossypium* species to test their levels of transferability. All the 23 *G. hirsutum*–derived EST-SSRs amplified products in (AD) 1 genome cultivars *G. hirsutum* L. cv *TM-1*, *CCRI 16*, and *CCRI 36*. Of the 23 EST-SSRs, 14 (60.9%) yielded microsatellite products from all 17 varieties, including 15 *Gossypium* species, whereas 9 (39.1%) amplified products from only a subset of the accessions, which ranged from 14 to 16 species. These results suggest that the EST-SSR markers derived from *G. hirsutum* produced a high rate of transferability across the *Gossypium* species. The transferability differed among genomes. The C1-, D1-, D5-, E2- and (AD)3-type genome species had lower transferability than others (Table 4).

**Table 4:** Transferability of G. *hirsutum*–derived EST-SSRs among different genomes in *Gossypium* species.

Genome	No. of SSRs amplified	Percentage amplified (%)	No. null amplified
A1	22	95.7	1
A2	23	100	0
C1	20	87.0	3
D1	21	91.3	2
D3	23	100	0
D4	23	100	0
D5	20	87.0	3
D6	23	100	0
D7	23	100	0
E2	21	91.3	2
(AD)1	23	100	0
(AD)2	23	100	0
(AD)3	20	87.0	3
(AD)4	23	100	0
(AD)5	22	95.7	1

## Discussion

Previous efforts in sequencing of upland cotton ESTs primarily utilized fibers or fiber-bearing ovules [30]–[32] and provided little or no information regarding flowering. In this study, 22,915 ESTs representing 14,373 unique sequences were identified from mRNA of upland cotton flower tissues, namely shoot apexes, flower squares, and flowers. The shoot apex samples cover the floral initiation period, and squares and flowers include the floral organ development period. This is the first and largest number of unique sequences from upland cotton flower-related tissues that includes all the developmental periods (floral meristem transition and flower development). These EST resources will be very useful for further studies, such as flowering-related gene discovery and molecular marker identification related to flower development of upland cotton. They will also help facilitate whole-genome sequencing and annotation.

Sequencing from a normalized cDNA library is a very cost-effective method for obtaining large-scale unique EST sequences and for gene discovery [33]. In our study, the cDNA library was normalized to subtract highly expressed genes and to isolate ESTs corresponding to rare or low-expression genes. EST assembly revealed a novelty rate of 62.7%, a redundancy rate of 37.3%, and 68.3% of unique sequences contained only one EST. These results clearly reflect the quality of the normalized library. They also demonstrate that this approach is a very cost-effective method to reduce the high variation among the abundant clones and increase the probability of sequencing rare transcripts.

In this study, all the unique ESTs were used to BLAST for functional annotations and categorization. Similarity searches of the EST sequences identified against those in the NCBI nr database revealed that of the 14,373 unique sequences, the great majority of unigenes identified (80.5%) had significant similarity with genes in plant species (including cotton). Higher similarities were found to *Ricinus*, *Vitis*, and *Populus*, which are all core eudicots and belong to the rosids, whereas cotton is *Malvales* and belongs to the eurosids II [34]. However, all these species have fully sequenced genomes and large EST databases, and the similarity does not necessarily reflect their phylogenetic proximity to upland cotton. The few hits (7.3%) to cotton sequences already available in GenBank suggest the lack of sequence information for this genus and reflect the value of the EST sequences generated in this study. Regarding the classification of known or putative functions, the largest proportion of the functionally categorized unigenes fell into three categories: “physiological processes” of the “biological process” category, “cell” of the “cellular component” category, and “binding activity” of the “molecular function” category. In our study, functional annotations and categorization of short ESTs were based only on the BLAST tool, and thus the results may be somewhat misleading for cases in which the hits were based on domain homologies rather than homology to orthologs [35].

Several putative flowering genes were identified from the results of functional annotations with other flowering plants. These include flowering determination genes such as *ZEITLUPE (ZTL)* and *FRIGIDA (FRI)*, which respond to environmental signals, and floral integrator genes *SUPPRESSOR OF OVEREXPRESSION OF CO1 (SOC1)* and *AP1 (APETALA1)*, which control floral organ development. In flowering plants, the transition from the vegetative to the reproductive phase is stimulated by environmental signals such as light and temperature. Day length is a major regulator of flowering time, and its measurement is taken to result from an external coincidence model based on a circadian clock [36], [37]. *ZEITLUPE (ZTL)* lengthens the free-running period of clock-controlled gene transcription and cell expansion, and it alters the timing of the day length–dependent transition from vegetative to floral development [38]. *FRIGIDA* (*FRI*) helps determine the natural variation in flowering time by perceiving cold [39]. The flowering determination process is regulated by distinct regulatory pathways. These input pathways are integrated by floral integrator genes, which are strong promoters of flowering [40]. The MADS-box transcription factor *SOC1* integrates multiple flowering signals derived from photoperiod, temperature, hormone, and age-related signals. The ABC model of flower organ identity shows that the A-, B-, and C-class organ identity genes specify identity to the four organ types, with A alone specifying sepals, A and B together specifying petals, B and C specifying stamens, and C alone specifying carpels [26]. The floral homeotic gene *AP1* encodes a putative transcription factor that acts locally to specify the identity of the floral meristem and to determine sepal and petal development [41]. The result of expression patterns of these putative homologs of *Arabidopsis* genes in upland cotton revealed that most are highly expressed in flowering tissues. The functions of these genes may be conserved in upland cotton flower development. However, only 34 flowering-related genes were identified from the best functional annotation; this may be because most of the best hits to the unigenes corresponded to unknown or putative proteins of plant species (*Ricinus*, *Vitis*, *Populus*, and so on) whose flower development is less characterized than that of the model plants *Arabidopsis* and rice. Thus, this library may be enriched with flowering homologs of other plant species, based on a BLASTx search and using *Arabidopsis* or rice flowering proteins as a query. Flowering genes Gh*SEP1* (JF271884), Gh*SEP2* (JF271885), and Gh*SEP3* (JF271886) were identified from our library using the *Arabidopsis* SEP proteins as a query to do a BLAST search, but they were not found using the unigenes annotation. This library will be a useful tool for cloning the full-length sequences of functional genes for further analysis in upland cotton.

Establishing a large EST library from upland cotton not only is the most efficient approach for gene discovery but also provides a critical resource for molecular marker development. Compared with all SSRs developed earlier and available in CMD, our EST resource identified 670 new perfect microsatellites having length >12 bp. The results suggest that trinucleotide repeats and the AT/TA motif are very common in coding sequences involved in flower development. It is possible that these trinucleotide motifs reside in the coding region and suppress frameshift mutations. AT repeats have been found in the untranslated regions of many species [42]–[44]. The differences in motifs may imply that different transcriptomes function as factors regulating gene expression, which could then lead to different expression characteristics of A- and D-subgenomes in tetraploid genomes. As the EST-SSR markers were derived from coding regions of DNA, they were more conserved and had a higher rate of transferability and polymorphism than genomic SSR markers [45]. In our study, the majority of the 23 EST-SSRs from upland cotton were highly transferable across cotton-related species. The transferability rate of these markers was high, suggesting that they can be used for comparative analysis of genetic diversity.

## Materials and Methods

### Plant material

Plants of upland cotton CCRI 36 (a short-season cotton) were field-grown during the summer of 2009. For the cDNA library construction, samples of tissues were isolated from shoot apexes (tip of the shoot containing the meristems as well as leaf initials), flower squares, and flowers. In short-season cotton such as CCRI 36, the shoot apical meristems begin to transform into inflorescence meristems when the two true leaves open completely. To study the entire flower development process, after the two true leaves had developed on each plant (15 days after sowing, DAS), shoot apex samples were collected at four different time points at 5-day intervals. Flower square samples were collected from 35 DAS to 55 DAS, also at 5-day intervals. Flower samples (including sepals, petals, stamens, and carpels) were isolated on the day of opening at 60 DAS. Samples for each stage and tissue type were pooled from at least two plants, frozen in liquid nitrogen, and stored at −80°C until further analysis.

### RNA extraction and cDNA library construction

Total RNA was extracted using a modified cetyltrimethyl ammonium bromide method, precipitated with diethyl pyrocarbonate water, and stored at −80°C. Equal amounts of total RNA from shoot apical meristems, squares, and flowers were mixed, and the RNA mixture was used to construct a full-length enriched cDNA library. The mRNA was isolated from total RNA with an Oligotex mRNA kit (Qiagen, Cat. No. 70022). With the material, a normalized and full-length cDNA library was prepared using the Creator SMART cDNA library construction kit (Clontech) and the TRIMMER-DIRECT cDNA Normalization kit (Evrogen) according to the manufacturers' protocols [46]. First-strand cDNA was synthesized using Superscript II reverse transcriptase in reactions containing SMART IV oligonucleotides (5′–AAGCAGTGGTATCAACGCAGAGTGGCCATTACGGCCGGG–3′) and CDS-3M adaptor (5′–AAGCAGTGGTATCAACGCAGAGTGGCCGAGGCGGCC(T)_20_VN–3′, where N = A, C, G or T; V = A, G or C). Double-stranded cDNA was amplified with long-distance PCR in a 100-µl reaction containing 10 µl 10×BD Advantage 2 PCR buffer, 2 µl dNTP mix (10 mM of each dNTP), 2 µl first-strand cDNA, 4 µl 5′ PCR primer (12 µM, 5′–AAGCAGTGGTATCAACGCAGAGT–3′), 2 µl 50×BD Advantage 2 polymerase mix, and 80 µl deionized water. The cycling parameters were 18 cycles of 95°C for 7 sec, 66°C for 20 sec, and 72°C for 4 min. The cDNA was analyzed using agarose gel electrophoresis and digested with duplex-specific nuclease followed by PCR. The conditions for the two-step amplification of the normalized cDNA were 15 cycles of 95°C for 7 sec, 66°C for 20 sec, and 72°C for 4 min for the first step. The second amplification was performed with 12 cycles of the same conditions. The normalized and amplified cDNA was digested with proteinase K and SfiI, size-fractionated with Chroma SPIN-400 columns, and analyzed with agarose gel electrophoresis. The size-fractionated cDNA was ligated into the plasmid vector pDNR-LIB and incubated at 16°C for 16 h. Electro-transformed *Escherichia coli* cells (DH10B) were spread on LB plates containing chloramphenicol (final concentration of 30 µg/ml). The plates were incubated at 37°C overnight, and colonies were picked and grown in 384-well plates at 37°C for 16–20 h and stored at −80°C.

### EST sequencing, editing, and assembly

Clones were randomly picked from LB agar plates supplemented with 30 µg/ml chloramphenicol. After the clones were manually picked, they were grown overnight in standard LB chloramphenicol medium, and the plasmids were isolated using the alkaline lysis method. Furthermore, plasmids from selected clones were sequenced using an ABI PRISM 3730xl automated DNA sequencer at the Sequencing Center of the Beijing Genomics Institute. Sequencing reactions were carried out from the 3′ end of the cDNA insert with the standard M13 reverse primer (5′-CAGGAAACAGCTATGAC-3′) using the ABI Prism BigDye Terminator Cycle Sequencing kit (Applied Biosystems). The parameters were 25 cycles of 96°C for 10 sec, 50°C for 6 sec, and 60°C for 4 min.

All sequences were clustered using the Phred/Phrap/Consed software package [47], [48]. Base calling was performed using Phred software (version phred_0.020425.c) with the quality cut-off set at Phred (Q13). Vector sequences were trimmed with Cross Match software (version 0.990329). The polyA tails of the sequences were eliminated using a program written by Beijing Genomics Institute. Any sequences with less than 100 quality bases after trimming were discarded. High-quality ESTs were aligned and assembled into contigs using Phrap software (version phrap_0.990329) when the criterion of a minimum identity of 95% over 40 bp was met. When an EST could not be assembled with others in a contig, it remained as a “singleton.” The contigs and singletons should thus correspond to sequences of unique genes.

To estimate the number of new ESTs generated in this work, all the assembled unigenes were compared with 351,954 cotton ESTs and 2,315 ETs in release 11.0 of the DFCI Cotton Gene Index (http://compbio.dfci.harvard.edu/cgi-bin/tgi/gimain.pl?gudb=cotton). A sequence is considered new if it has at least 10% of the sequence and is less than 95% identical to any other EST or unigene in the public EST database.

### Unigene functional annotation and functional categorization

All unique sequences were used to search for putative ORFs with getorf software EMBOSS-4.1.0 [49], and the longest sequences were used for functional analysis. Sequence similarity searches were performed using the BLASTx programs and were then compared to a variety of databases including NCBI nt, NCBI nr (http://www.ncbi.nlm.nih.gov), and SwissProt (http://www.expasy.org/sprot/). The cut-off E-value of the BLAST searches was 1e-5. To assign GO terms, unique sequences were searched using InterProScan software against the annotated sequences of the GO database (http://www.geneontology.org/). The distribution of GO terms in each of the main ontology categories of biological processes, cellular components, and molecular functions was examined.

### Flowering homolog identification in upland cotton and expression analysis

The homologs of plant flowering-related protein sequences were identified from the EST function annotation. The genes were identified if the top-ranked EST hits were flowering-related proteins of other species. The gene expression in cotton tissues was analyzed by qRT-PCR using the fluorescent intercalating dye SYBR-Green in an ABI PRISM 7500 Sequence Detection System (Applied Biosystems). The qRT-PCR was carried out using the SYBR GREEN PCR Master mix (Applied Biosystems), and the cDNA was reverse-transcribed from RNA samples from root, stem, leave, shoot apex, square, flower, fiber, and ovule. A three-step RT-PCR procedure was performed in all experiments. Expression levels were calculated relative to the constitutively expressed gene encoding the 18S ribosomal RNA (18S) and the reference sample root. Normalization was carried out using the comparative Ct method (Applied Biosystems). As a result, the relative expression level of the target was normalized to an endogenous reference.

### Isolation of Gh*SEP*s and sequence analysis

*Arabidopsis SEP* genes were used as a query to tBLASTn search against the cDNA library. The clones identified were sequenced from two directions with the internal primers. Nucleotide and amino acid sequences were analyzed using DNAstar and GeneDoc. For phylogenetic analysis, genes and the homologs of other plants were aligned with the ClustalW program (http://www.ebi.ac.uk) followed by the neighbor-joining method analysis using MEGA 4.1 [50]. The expression patterns were detected by qRT-PCR as described above.

### EST-SSR identification and primer design

To assess the potential of the newly developed EST-SSRs, 14,373 unigenes were examined with the web-based software SSRIT [51]. The SSR motifs, with repeat units of more than six in dinucleotides, four in trinucleotides, and three in tetranucleotides, pentanucleotides, and hexanucleotides were used for the search criteria. To identify the new SSR markers, all the developed EST-SSRs were compared with the CMD cotton sequences using BLASTn (E-value cutoff 1e-05). The SSR-containing ESTs were then identified as candidates for SSR marker development if they had sufficient sequences on both sides of the SSR repeats for primer design. Primer 3 software was used to design the primers [52]. The following parameters were used: primer length 15–22 bp, with 20 bp as the optimum; primer GC%  = 40–70%, with the optimum value being 50%; primer T_m_ 50–60°C, and product size range 100–300 bp.

### EST-SSR screening and polymorphism survey

Twenty-five of the primers were randomly chosen and synthesized by Invitrogen, Shanghai PR China. Total DNA was isolated from the leaf according to the cetyltrimethyl ammonium bromide method [53]. PCR amplification was conducted in 25-µl reactions containing 50 ng of template DNA, 2.5 mM MgCl_2_, 2.5 µl 10×PCR buffer, 0.5 mM each primer, 0.5 U Taq DNA polymerase, and 2.5 mM dNTPs. The PCR cycling profile was 94°C for 5 min, 30 cycles at 94°C for 45 s, 60°C for 45 s, 72°C for 45 s, and a final extension at 72°C for 10 min. The quality of the PCR product was checked by mixing it with an equal volume of loading buffer and then visualizing the band on a 2.0% agarose gel in 1× TBE buffer at 100 W for 120 min. All primers were first tested in *G. hirsutum* L. cv CCRI 36 to determine whether they could amplify the target DNA. The primers that were successful in giving products were then used to assess transferability in 17 *Gossypium* species (Table 5). Transferability was calculated as the amounts of EST-SSR products amplified in *Gossypium* species [54].

**Table 5:** *Gossypium* species used in this study.

No.	Species name	Abbreviation	Ploidy	Genome	Cultivated/Wild
1	*G. herbaceum* var. *africanum*	GHERB	2×	A1	Cultivated
2	*G. arboretum* L. *cv Feng*×*ian*	GARBO	2×	A2	Cultivated
3	*G. sturtianum Willis*	GSTUR	2×	C1	Wild
4	*G. thurberi Tod.*	GTHUR	2×	D1	Wild
5	*G. davidsonii Kell.*	GDAVI	2×	D3-d	Wild
6	*G. aridum (Rose & Standl.) Skov.*	GARID	2×	D4	Wild
7	*G. raimondii Ulbr.*	GRAIM	2×	D5	Wild
8	*G. gossypioides (Ulbr.) Standl.*	GGOSS	2×	D6	Wild
9	*G. lobatum Gentry*	GLOBA	2×	D7	Wild
10	*G. somalense (Gurke) Hutch.*	GSOMA	2×	E2	Wild
11	*G. hirsutum* L. cv *TM-1*	TM-1	4×	(AD)1	Cultivated
12	*G. hirsutum* L. cv *CCRI 36*	CCRI36	4×	(AD)1	Cultivated
13	*G. hirsutum* L. cv *CCRI 16*	CCRI16	4×	(AD)1	Cultivated
14	*G. barbadense* L. var. *Giza 75*	GIZA75	4×	(AD)2	Cultivated
15	*G. tomentosum Nuttall* e× *Seemann*	GTOME	4×	(AD)3	Wild
16	*G. mustelinum Miers* e× *Watt*	GMUST	4×	(AD)4	Wild
17	*G. darwinii Watt*	GDARW	4×	(AD)5	Wild

Supporting Information.

## Supporting Information

Table S1 Upland cotton unigenes annotated to flower development genes and best BLASTx hits to other species.
(DOC)

Table S2 Microsatellite markers developed for upland cotton.
(XLS)

Table S3 Summary of EST-SSR primers and repeat motifs.
(DOC)
