# Supplementary material for: Generation of ESTs for Flowering Gene Discovery and SSR Marker Development in Upland Cotton
Source: PLoS One. 2011 Dec 6;6(12):e28676. doi: 10.1371/journal.pone.0028676 (PMC3232235; doi:10.1371/journal.pone.0028676)
Supplement: Table S1 — Upland cotton unigenes annotated to flower development genes and best BLASTx hits to other species. (DOC) [file pone.0028676.s001.doc]

**Table S1. Upland cotton unigenes annotated to flower development genes and best BLASTx hits to other species.**

|  | **Best BLASTx match** | | | | |
| --- | --- | --- | --- | --- | --- |
| **Cluster ID** | **Protein name** | **Accession** | **Biological function** | **E-value** | **Identity** |
| HO110721 | *PHYE* | P55004 | Red light photoreceptor [*Ipomoea nil*] | 2.00E-16 | 57 |
| HO107566 | *CRY1* | Q70AD6 | Blue light photoreceptor [*Spalax judaei*] | 5.00E-10 | 62 |
| Contig3480 | *ZTL* | Q94BT6 | F-box protein/blue light photoreceptor [*Arabidopsis thaliana*] | 1.00E-110 | 94 |
| Contig2859 | *FYPP3* | Q8LSN3 | Ser/Thr-specific protein phosphatase 2A [*Pisum sativum*] | 9.00E-58 | 96 |
| HO102429 | *CCA1* | P92973 | Myb domain TF [*Candida glabrata*] | 8.00E-52 | 52 |
| Contig2307 | *GI* | Q9AWL7 | protein binding [*Oryza sativa* subsp. *Japonica*] | 7.00E-17 | 72 |
| Contig2950 | *PRR5* | Q6LA42 | Pseudo-response regulator [*Arabidopsis thaliana*] | 3.00E-22 | 54 |
| HO090503 | *CO* | Q39057 | putative zinc finger TF [*Arabidopsis thaliana*] | 7.00E-11 | 42 |
| Contig5671 | *COL3* | Q9SK53 | putative zinc finger TF [*Arabidopsis thaliana*] | 1.00E-32 | 51 |
| HO106939 | *FD* | Q84JK2 | bZIP TF [*Arabidopsis thaliana*] | 2.00E-12 | 61 |
| HO095946 | *COP1* | P43254 | E3 ubiquitin ligase [*Arabidopsis thaliana*] | 3.00E-60 | 86 |
| Contig1424 | *ADG1* | P52417 | glucose-1-phosphate adenylyltransferase activity [*Vicia faba*] | 6.00E-76 | 92 |
| Contig4813 | *COL2* | Q96502 | putative zinc finger TF [*Arabidopsis thaliana*] | 4.00E-20 | 62 |
| Contig6357 | *COL1* | O50055.1 | putative zinc finger TF [*Arabidopsis thaliana*] | 2.00E-18 | 57 |
| Contig2720 | *SOC1* | O64645 | MADS box TF [*Arabidopsis thaliana*] | 1.00E-73 | 68 |
| Contig1211 | *FRI* | XP_002517136 | enhancer of FLC [*Ricinus communis*] | 3.00E-66 | 69 |
| Contig5960 | *FES1* | P37841 | CCCH zinc finger protein [*Solanum tuberosum*] | 9.00E-72 | 91 |
| Contig854 | *VIP3* | Q9M4C4 | RNA polymerase 2 associated factor -like [Avena fatua] | 2.00E-37 | 69 |
| Contig2608 | *VIN3* | Q9FIE3 | PHD domain protein [*Arabidopsis thaliana*] | 7.00E-31 | 64 |
| Contig3162 | *VRN1* | Q8L3W1 | DNA binding protein [*Arabidopsis thaliana*] | 9.00E-43 | 79 |
| HO109998 | *EMF2* | Q8L6Y4 | Polycomb group protein [*Arabidopsis thaliana*] | 1.00E-70 | 84 |
| HO106504 | *SPY* | Q96301 | O-linked N-acetylglucosamine transferase [*Arabidopsis thaliana*] | 6.00E-71 | 60 |
| HO108072 | *MSI1* | O22466 | WD40 protein [*Solanum tuberosum*] | 1.00E-59 | 95 |
| HO104079 | *SKB1* | Q8GWT4 | Type 2 protein arginine methyltransferase [*Arabidopsis thaliana*] | 1.00E-16 | 67 |
| HO090425 | *FLD* | Q9CAE3 | component of histone deacetylase complex [*Arabidopsis thaliana*] | 2.00E-50 | 61 |
| Contig2755 | *SVP* | Q9FVC1 | MADS box TF [*Arabidopsis thaliana*] | 5.00E-13 | 79 |
| Contig4234 | *AP1* | Q41276 | MADS box TF [*Arabidopsis thaliana*] | 1.00E-48 | 60 |
| HO099311 | *CAL* | Q39081 | MADS box TF [*Arabidopsis thaliana*] | 3.00E-32 | 49 |
| HO098204 | *UFO* | Q39090 | ubiquitin-protein ligase activity [*Arabidopsis thaliana*] | 3.00E-42 | 62 |
| Contig6300 | *FUL* | Q42429 | MADS box TF [*Solanum tuberosum*] | 6.00E-78 | 64 |
| Contig3214 | *WUS* | Q6YBV1 | Homeobox TF [*Arabidopsis thaliana*] | 2.00E-41 | 64 |
| HO097163 | *HUA1* | Q941Q3 | RNA processing [*Arabidopsis thaliana*] | 2.00E-40 | 75 |
| Contig7670 | *GLO* | Q03416 | DNA binding [*Nicotiana tabacum*] | 1.00E-25 | 54 |
| Contig5083 | *AG* | Q40872 | DNA binding [*Panax ginsen*] | 1.00E-91 | 75 |
