# Supplementary material for: Generation of ESTs for Flowering Gene Discovery and SSR Marker Development in Upland Cotton
Source: PLoS One. 2011 Dec 6;6(12):e28676. doi: 10.1371/journal.pone.0028676 (PMC3232235; doi:10.1371/journal.pone.0028676)
Supplement: Table S3 — Summary of EST-SSR primers and repeat motifs. (DOC) [file pone.0028676.s003.doc]

**Table S3. Summary of EST-SSR primers and repeat motifs.**

| **Marker name** | **SSR motif** | **Primer sequence (5′-3′)** | **T_m_**  **(°C)** | **Expected product (bp)** | |
| --- | --- | --- | --- | --- | --- |
| CCRI001 | (AGCA)3(GTTT)6 | F:TCGCATATGAGAAATCAAGCA  R:TTACCGGATGCTACAGACCC | 59.7 | 216 |  |
| CCRI002 | (TTAT)4 | F:CAATGGCTGCCCATATGTAA  R:CTGGTCACCGTTCTCGTTCT | 59.8 | 279 |  |
| CCRI003 | (CAT)12 | F:CAGCGAGTGATCCTTGAACA  R:AAGGCCACTTCCAATCTCAA | 59.8 | 282 |  |
| CCRI004 | (CATA)4 | F:GGGCAATAAGGTACACCGAA  R:TCGCCTTTACCAAAACCAAC | 59.9 | 279 |  |
| CCRI005 | (AAAAC)4 | F:AACTCAAAGCAATGAGCCGT  R:CCTGACACACGCTGCATTAT | 59.8 | 188 |  |
| CCRI006 | (TCA)5(ATC)4  (TCTTCT)3(TTC)6 | F:CACTGTTTGCATCCCACAAC  R:CTACTGGACGGAATCGGTGT | 60.0 | 295 |  |
| CCRI007 | (CATA)3(AT)  6(AGAT)3 | F:TCTAAGCAACAGGCACCCTT  R:CCATGAAGGATACAATGGGG | 59.9 | 256 |  |
| CCRI008 | (TACAG)4 | F:AGTCCAATCTTAGAGCCCCC  R:TGGGAGAGAGGCAACGTATT | 59.6 | 173 |  |
| CCRI009 | (GGA)5 | F:GGAAAACCCATACGAGGGAT  R:CGTCGAAATCGAAGGAAAAG | 59.9 | 182 |  |
| CCRI010 | (TGA)5 | F:TATGGCCTGATTGACCTTCC  R:CAAGAATGTTGCTTGCCTGA | 59.9 | 128 |  |
| CCRI011 | (AATA)3(AACA)5 | F:ATGCTGACATCATCCCTTCC  R:ATGGAATTTCTTGCATTGCC | 59.9 | 244 |  |
| CCRI012 | (GA)18(CAGCA)4 | F:CGTGATAGGATTGGAGGAGG  R:ACGAGTCTACCCGTACCGTG | 59.8 | 286 |  |
| CCRI013 | (CTG)5 | F:ACACTAGGCCGACCAATGAC  R:GATCGGAGGGGCAGATTTAT | 60.1 | 248 |  |
| CCRI014 | (ATC)5 | F:GAATCGGAAGACAGGCGTAA  R:CCCTATAAGTGCCTGCAACA | 59.5 | 216 |  |
| CCRI015 | (AAT)4(TA)7 | F:AGGCAAGTATTGGCCACAAG  R:CCGGTGATTCTTGATGCTTT | 60.1 | 293 |  |
| CCRI016 | (CT)15 | F:AATTCTGCAGGACTGGATGG  R:AAATGTGGAAGGAGCACAGG | 60.1 | 288 |  |
| CCRI017 | (ACAAA)5 | F:CGCTACAAGTCCGAAACATCT  R:ATTTATGGGGCCACTTGTGA | 59.8 | 143 |  |
| CCRI018 | (ATCCCT)5 | F:CTTTCCCTTCCCCTATCTCG  R:AGTTGGTGACTGGGTTTTCG | 60.0 | 191 |  |
| CCRI019 | (GCA)5 | F:CCTTTAACATCGGGGCACTA  R:CAAGCCAAGTTGTTGCTGAA | 60.0 | 253 |  |
| CCRI020 | (TGA)4(GAT)9 | F:GACCTTCAGCTTGCTTTGCT  R:TTGAACAAGTCCGGGAAGTC | 59.9 | 210 |  |
| CCRI021 | (AAAAAG)5 | F:TGCACCAAATTGCCATATGTA  R:AAACGTGTCCGCTTTGTAGG | 60.0 | 236 |  |
| CCRI022 | (AT)7 | F:CTAAATCGTTGCTTGCGTGA  R:GGACTTCTCCCTCAAAATGGA | 60.2 | 290 |  |
| CCRI023 | (AT)8 | F:GTTTGCTCCACCATCGAAAT  R:ACCGACAGATGTGGGGAATA | 60.1 | 205 |  |
| CCRI024 | (ATAC)6 | F:TCGCATAGTCACATCCTTGC  R:TTTCATGGGTTCTTCCTGCT | 59.8 | 197 |  |
| CCRI025 | (AT)8 | F:AACCAGGACAAACATAGGCG  R:GGAGGTTTTGTGGATCCCTT | 60.1 | 136 |  |
